# Effect of matrix properties on transmission and reflectance mode division-of-focal-plane Stokes polarimetry

**DOI:** 10.1117/1.JBO.28.10.102902

**Published:** 2023-07-11

**Authors:** Leanne E. Iannucci, Matthew B. Riak, Ethan Meitz, Matthew R. Bersi, Viktor Gruev, Spencer P. Lake

**Affiliations:** a Washington University in St. Louis, McKelvey School of Engineering, Department of Biomedical Engineering, St. Louis, Missouri, United States; b Washington University in St. Louis, McKelvey School of Engineering, Department of Mechanical Engineering and Materials Science, St. Louis, Missouri, United States; c University of Illinois Urbana-Champaign, Department of Electrical and Computer Engineering, Champaign, Illinois, United States; d Washington University in St. Louis, School of Medicine, Department of Orthopaedic Surgery, St. Louis, Missouri, United States

**Keywords:** polarized light, collagen, biomedical imaging, anisotropy, birefringence

## Abstract

**Significance:**

Division-of-focal-plane Stokes polarimetry is emerging as a powerful tool for the microstructural characterization of soft tissues. How individual extracellular matrix (ECM) properties influence polarimetric signals in reflectance or transmission modes of quantitative polarized light imaging (QPLI) is not well understood.

**Aim:**

We aimed to investigate how ECM properties affect outcomes obtained from division-of-focal-plane polarimetric imaging in reflectance or transmission modes.

**Approach:**

Tunable collagen gel phantoms were used to modulate ECM properties of anisotropy, collagen density, crosslinking, and absorber density; the effects of degree of linear polarization (DoLP) and angle of polarization (AoP) on polarimetry outcomes were assessed. A model biological tissue (i.e., bovine tendon) was similarly imaged and evaluated using both reflectance and transmission modes.

**Results:**

Reflectance QPLI resulted in decreased DoLP compared with transmission mode. A 90 deg shift in AoP was observed between modes but yielded similar spatial patterns. Collagen density had the largest effect on outcomes besides anisotropy in both imaging modes.

**Conclusions:**

Both imaging modes were sufficiently sensitive to detect structural anisotropy differences in gels of varying fiber alignment. Conclusions drawn from phantom experiments should carry over when interpreting data from more complex tissues and can help provide context for interpretation of other Stokes polarimetry data.

## 1 Introduction

Collagen is the most abundant extracellular matrix (ECM) protein in the human body,^1^ and it is organized hierarchically to provide efficient load-bearing ability to biological tissues. In fibrous connective tissues, collagen fibers contain sinusoidal crimp patterns that enable non-linear mechanical compliance when loaded.^2^ Additionally, the fibers themselves are highly aligned in the direction of dominant loading, which results in high tensile but low flexural fiber stiffness.^3^^,^^4^ Therefore, the orientation and alignment of the collagen fiber network influence soft tissue mechanics and biological function. Indeed, the disruption of collagen fiber alignment can lead to aberrant cellular responses, which contribute to pathology and eventual tissue failure. For this reason, it is essential to visualize the collagen fiber architecture in soft biological tissues to detect if sub-failure structural damage has occurred in different mechanical environments. Without a comprehensive and dynamic visualization technique, it can also be difficult to evaluate the efficacy of regenerative approaches on tissue healing and ECM remodeling. Thus there is a clear need for an imaging modality that can spatially resolve the collagen fiber structure while also providing temporal resolution to elucidate dynamic structure–function relationships.

Collagenous soft tissues exhibit birefringence due in part to their structural anisotropy, enabling polarized light-based imaging techniques to probe tissue structure endogenously.^5^ When light interacts with birefringent materials, a phase delay is induced between the perpendicular components of the electric field of the incident light, which imposes ellipticity in the light’s polarization state.^6^ The amount of retardation of the incident light is directly related to the degree of birefringence and thus the collagen fiber alignment of the material.^7^ By measuring the change in the polarization state of light, the structural information about the tissue of interest can be extracted. This principle is foundational to most polarized light-based microscopy and imaging techniques.^8^ Additionally, because the structural integrity and anisotropy of biological tissues change in periods of disease^7^ or in response to loading,^6^^,^^9^ non-destructive polarimetry principles can be used to explore pathology and dynamic tissue responses.

Polarimetry systems are typically based on either Mueller or Stokes formalisms.^10^^,^^11^ Mueller systems aim to directly characterize the polarimetric properties of a tissue of interest. However, Mueller systems require multiple illumination states for a single data acquisition point and thus can suffer from low temporal resolution and more involved post-processing efforts. Stokes polarimetry, on the other hand, aims to indirectly infer tissue properties by characterizing only the polarization state of light after interaction with a sample of interest. Stokes systems are much simpler, require minimal instrumentation, and are capable of real-time imaging in a “single shot” manner. Further, with recent commercialization of polarimetric technologies such as the Sony Polarsens,^12^ division-of-focal-plane approaches for Stokes polarimetry are becoming increasingly common. The latter has emerged as a powerful technique in the biomedical space and has been widely used in the characterization of microstructural properties of musculoskeletal soft tissues.^6^^,^^13^^–^^19^

There are several configurations, or modes, that Stokes polarimetry systems can take that greatly affect signal interpretation.^10^ Transmission mode imaging is typically performed on *ex vivo* samples that have been excised and thinned. Imaging in this mode involves the collection of mostly forward-scattered photons that provide thickness-averaged information of light interactions within the tissue, thus leaving no room for extraction of depth-dependent signals.^20^ Reflectance mode polarimetry is more suitable for non-contact, *ex vivo* and *in vivo* imaging of bulk tissues because photons collected in this mode are backscattered and have undergone substantially more scattering events compared with their transmission mode counterparts.^21^

Despite its widespread usage, the full interpretation of results obtained from polarimetry-based techniques remains poorly defined. Output polarimetry values have been interpreted as being due to several different ECM properties: structural anisotropy, collagen crosslinking, or even differences in the molecular structure of the collagen type (e.g., type I versus type III collagen).^22^^,^^23^ There is likely some amount of signal contribution from each of these factors, although it is difficult to decouple these effects in a complex biological setting. To our knowledge, there has only been one systematic study probing signal differences obtained during biomedical polarimetry of biological tissues based on varying ECM properties.^24^ However, this study only evaluated the effects of collagen density and crosslinking across a small range of values and only considered a Mueller matrix polarimetry technique in transmission mode.

In this study, we aim to leverage the tunability and relative simplicity of a collagen gel model system to explore how both (1) collagen fiber alignment and (2) isotropic ECM properties (e.g., collagen density, crosslinking density, and absorber density) affect outcomes in a division-of-focal-plane Stokes polarimetry system. We also explore differences in transmission and reflectance mode imaging, as we hypothesize that the measured ECM properties may be uniquely affected by differences in the light–tissue interactions associated with photons collected in each imaging mode. Finally, we image a representative biological tissue [i.e., bovine flexor tendon (BFT)] to assess how fundamental observations from the collagen gel model can be extended to the analysis of more physiologically relevant collagenous soft tissues.

## 2 Materials and Methods

### 2.1 Quantitative Polarized Light Imaging

Quantitative polarized light imaging (QPLI) is the Stokes polarimetry-based imaging technique leveraged in this study. In brief, the QPLI system consisted of a white LED illuminator (SugarCUBE, Ushio America, Cypress, California, United States; 400 to 700 nm) directed using a semi-rigid fiber optic light guide through a polarization state generator made up of a right-handed circular polarizing polymer film (CP42HER; Edmund Optics, Barrington, New Jersey, United States). The polarization state analyzer and detector were integrated in a single unit as a division-of-focal-plane polarimeter. The polarization sensor was fabricated by Sony (PolarSens, IMX250MZR Polar-Mono) and is onboard a 5.0 MP, 75 fps FLIR BlackFly S USB3 camera module (BFS-U3-51S5P-C; Teledyne FLIR, Wilsonville, Oregon, United States).

**Fig. 1:**
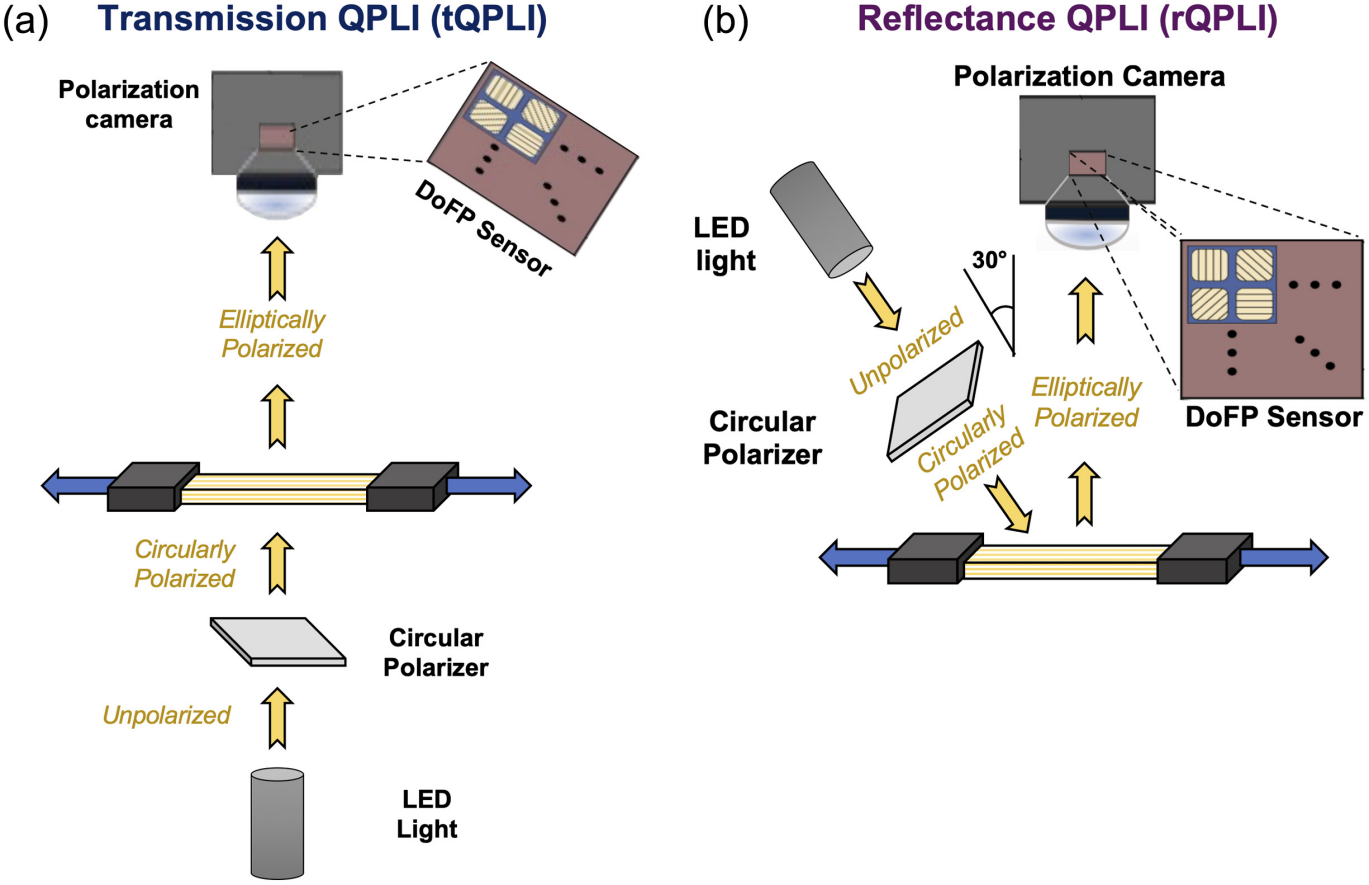
Schematics of the QPLI system in (a) transmission mode (tQPLI) and (b) reflectance mode (rQPLI).

In transmission mode QPLI [tQPLI; Fig. 1(a)], the light guide was a Dolan-Jenner fiber optic backlight (39-826; Edmund Optics, Barrington, New Jersey, United States) that was configured to transmit light through the sample of interest for a sample illuminance of 260 lux, in-plane with the DoFP detector. In reflectance mode QPLI [rQPLI; Fig. 1(b)], a single branch semi-rigid light guide (54-210; Edmund Optics, Barrington, New Jersey, United States) was directed at the sample at an angle of 30 deg from the surface normal of the sample of interest, resulting in a sample illuminance of 280 lux. The DoFP detector was positioned directly above the sample (in-line with the surface normal). This configuration avoids the collection of any specularly reflected light while maximizing signal-to-noise.^25^

High resolution images (2448×2048  pixels) were acquired by the DoFP polarimeter at 20 fps via an in-house developed software suite and saved in an h5 file format.^26^ We did not perform additional calibration on the polarization data obtained from the polarimeter. The optical properties of the Sony PolarSens (spatial uniformity of ∼0.5%, extinction ratio>300) are superior compared with properties of the first polarization camera published in the literature^27^ (spatial uniformity of 8%, extinction ratio of over ∼50), where calibration was prominent due to manufacturing defects of the pixelated polarization filters. Although calibration should be included as part of the image processing toolbox for high accuracy polarization imaging, current experimental configurations either lack the optical accuracy (i.e., producing polarization fields with <0.5% uniformity across the imaging array and extinction ratios of more than 1000) or are prohibitively expensive to construct. Post-processing of the raw polarization data was performed using a custom MATLAB script. In brief, the sensor captured repeats of a 2×2 array (superpixel) of oriented linear polarizing filters over each pixel oriented at θ=0  deg, 45 deg, 90 deg, and 135 deg. The raw polarization image data were interpolated using bilinear algorithms, which improves spatial resolution and accuracy of the reconstructed polarization information.^28^ The first three Stokes parameters ($S_{0},S_{1},S_{2}$), and subsequently the degree of linear polarization (DoLP) and angle of polarization (AoP), were calculated for each superpixel across the entire field of view, as described previously.^6^

Additionally, user-selected sample-spanning region of interest (ROI) masks were generated. For each frame, the average DoLP (AVG DoLP) and standard deviation of the AoP (STD AoP) were calculated for the defined ROI.^6^ AVG DoLP is a measure of the strength of collagen fiber alignment and is determined from the average of all DoLP values within the ROI. Additionally, STD AoP is calculated as the standard deviation of all AoP values within the ROI, thereby representing the relative variance (spread) of collagen fiber orientation directions.

### 2.2 Tissue Phantoms of Varying Collagen Fiber Alignment

#### 2.2.1 Phantom generation

Fibroblast-seeded collagen gel tissue phantoms were used in this study as they are a widely-used model system that allows for the generation of simple biological tissue analogs with controllable collagen fiber alignment.^29^^–^^32^ To generate the collagen gels, neonatal human dermal fibroblasts (nHDFs) (ATCC, Inc., Manassas, Virginia, United States) were expanded out to passage 4 in complete culture media [Dulbecco’s Minimum Essential Medium (DMEM) supplemented with 10% fetal bovine serum (FBS) and 1% penicillin/streptomycin]. Type I collagen was isolated from tail tendons of Long Evans rats using previously established methods,^33^ dissolved in 0.1% acetic acid at a stock concentration of 2.25  mg/mL, and then stored at 4°C until use. At the time of collagen gel generation, stock collagen was brought to pH 7.0 and 300 mOsm by adding 1 N NaOH and phosphate buffered saline. Neutralized collagen was then mixed with nHDFs suspended in complete media for a final concentration of 1 million cells/mL in 1.5  mg/mL collagen (4:1:1 ratio of stock collagen:cell suspension:neutralization solution).

**Fig. 2:**
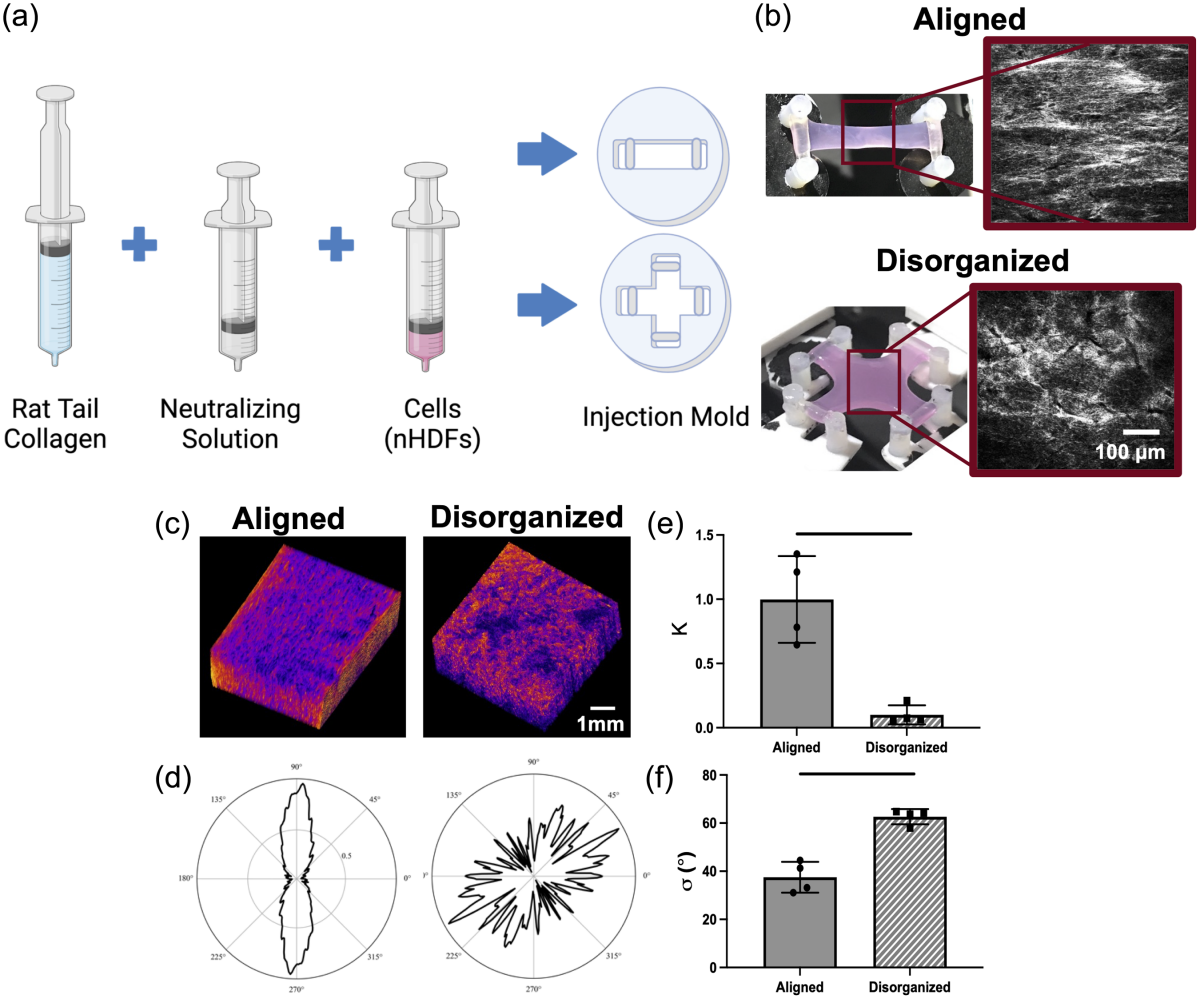
Fabrication and validation of collagen tissue phantom microstructure. (a) Schematic of collagen gel fabrication process. (b) Images of collagen gel phantoms after 7 days of static culture. Inset is a representative SHG image taken from the center of each gel type. (c) 3D renderings of OCT image stacks taken from the center of each phantom after static culture. The central 5 image slices from the OCT stack were taken for further quantitative analysis. (d) Polar plot of fiber orientations extracted from FFT-based image processing. Data were fit to a Von Mises distribution to obtain values of (e) K, fiber concentration and (f) σ, variance of fiber orientations. Unpaired t-test compared groups (n=4), where lines indicate p<0.05. Plots are mean ± SD.

The cell–gel mixture was then injected into one of two 2.5 mm thick Teflon mold types to designate the final type of collagen fiber alignment after the culture period: aligned or disorganized. Aligned molds were rectangular in shape and disorganized molds were cruciform [Fig. 2(b)]. Borosilicate rods within the ends of the molds provided static mechanical boundary conditions during culture. Gels cast in each mold type were allowed to polymerize at 37°C for 30 min, then covered in DMEM with 10% FBS and 1% penicillin/streptomycin, and static cultured for 7 days. Over this culture duration, gels in aligned molds generated highly anisotropic collagen alignment, whereas disorganized molds produced phantoms with isotropic alignment in the center region (Fig. 2). The microstructural properties of each gel were validated using two optical imaging modalities: (1) second harmonic generation (SHG) imaging [Fig. 2(b)] and (2) optical coherence tomography (OCT) [Figs. 2(c)–2(f)].

#### 2.2.2 Microstructural validation

For SHG validation [Fig. 2(b)], phantoms were imaged using a deep *in vivo* explorer Leica Sp8 Multiphoton Microscope. A spectraphysics pulsed infrared laser was tuned to 880 nm excitation and a non-descanned HyD detector collected emitted SHG signals at 430 to 450 nm. Representative SHG images were taken at the centers of each gel, and the microstructure was qualitatively evaluated.

For OCT evaluation [Figs. 2(c)–2(f)], phantoms were imaged using a Thorlabs Ganymede Series OCT system with a center wavelength of 930 nm, providing an imaging depth of up to 2.2 mm in water at an axial resolution of 4.1  μm and lateral resolution of 8  μm. Once imaged, all z-stacks were processed using a standardized image processing pipeline. The five centermost slices from the z-stack were extracted and subjected to a MATLAB-based coherence-enhancing diffusion filter.^34^^,^^35^ This filter enhances “flow-like” structures, such as collagen fibers, blood vessels, and elongated cells, while simultaneously reducing background noise. After being processed, filtered images were analyzed using the FiberFit python-based software suite.^36^ In brief, a fast Fourier transform (FFT) was performed and a periodic von Mises distribution was fit to the resulting fiber angle distribution to calculate two outcome parameters: (1) a measure of anisotropy known as the fiber concentration k, where larger values indicate a greater degree of alignment, and (2) the variance in fiber orientation σ, where larger values indicate more distributed fibers. As k and σ are analogous to values of AVG DoLP and STD AoP, respectively, these outcome measures were extracted for each image. Unpaired t-tests were used to statistically evaluate differences in k and σ between alignment groups, where p<0.05 was considered to be statistically significant.

#### 2.2.3 Experimental design and statistical testing

After maturation, gels of both alignment groups were removed from culture and subjected to simultaneous QPLI and dynamic mechanical testing (n=6). All gels underwent a 0.01 N preload on all arms and were imaged once by rQPLI and once by tQPLI; gels were randomized as to which mode would occur first. QPLI outcomes of AVG DoLP and STD AoP were calculated for each ROI and were statistically analyzed via paired t-tests between imaging modes within a single alignment type. Representative color maps for DoLP and AoP at baseline for each gel in each imaging mode were generated.

### 2.3 Tissue Phantoms of Varying Extracellular Matrix Properties

#### 2.3.1 Phantom fabrication

**Fig. 3:**
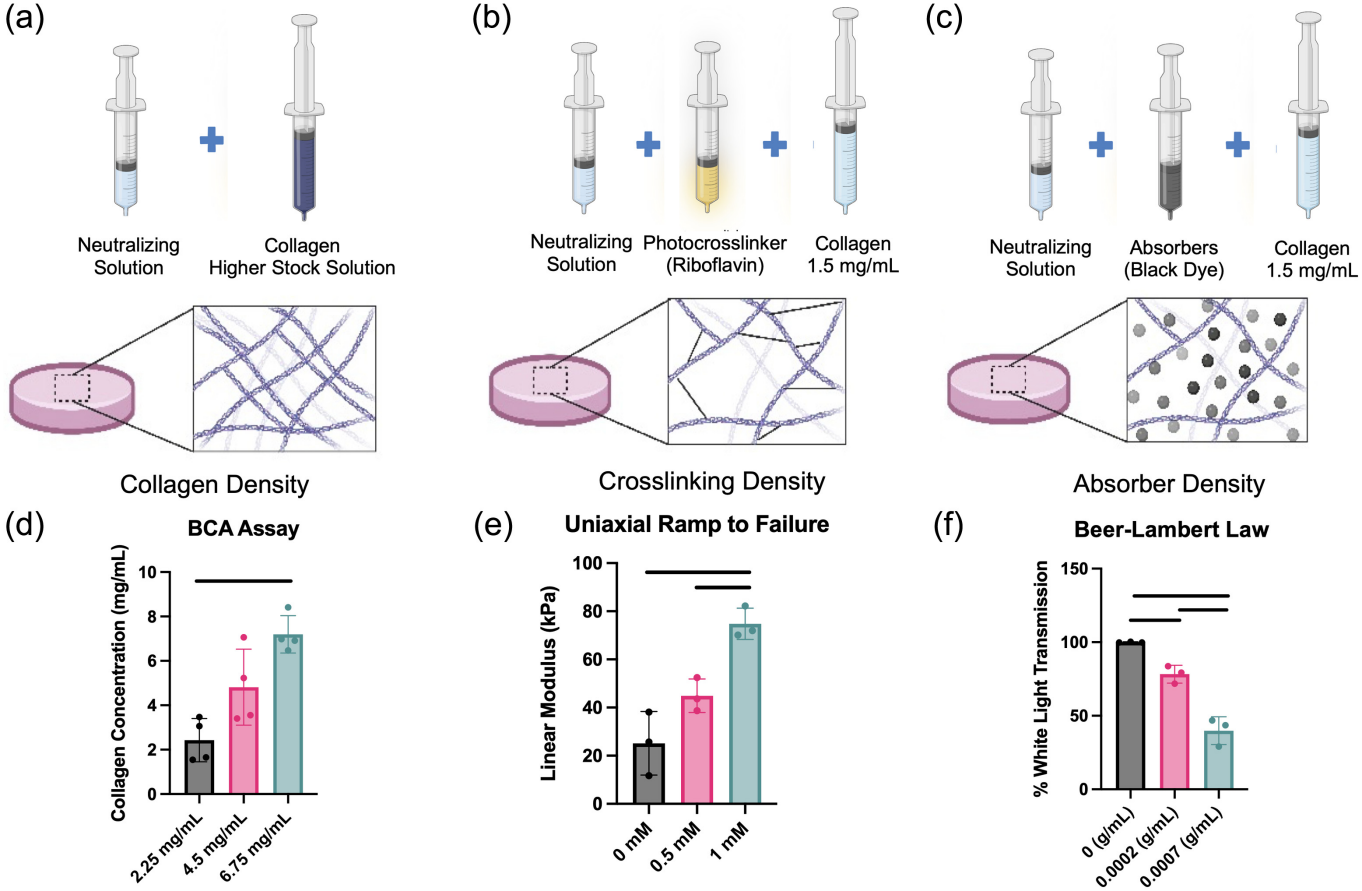
Abrication of collagen tissue phantoms with modified ECM properties of (a) collagen density, (b) crosslinking density, and (c) absorber density. (d) Collagen density was secondarily validated with the BCA assay (n=3). (e) Crosslinking density was validated by measuring the linear modulus in a uniaxial ramp-to-failure test (n=3). (f) Absorber density was validated by measuring the % of white light transmission at the chosen density compared with controls using the Beer–Lambert law (n=3). One way ANOVA with Tukey’s HSD *post hoc* tests were used for statistical evaluation, lines indicate p<0.05. Plots are mean ± SD.

Collagen gel tissue phantoms were fabricated similar to the anisotropic varying tissue phantoms but without the cellular component (Fig. 3). After neutralization, the collagen mixture was cast into a custom made optically inert injection mold that was fabricated using dyed PDMS,^37^ plasma treated, and covalently bonded to a glass plate. There were eight wells per mold with a diameter of 10 and 5 mm in depth. After injection molding, the collagen gels were placed at 37°C for 30 min to allow for collagen fibril self-assembly and polymerization. Experimental groups were created by altering steps or adding components to the collagen gel fabrication procedure.

#### 2.3.2 Varying collagen density

To create collagen gels with altered densities [Fig. 3(a)], the stock collagen solution used was 2.25  mg/mL (control) or was changed to be either 4.5 or 6.75  mg/mL. Concentrations of stock solutions were confirmed via bicinchoninic acid (BCA) assay against a type I collagen standard [Fig. 3(d)]. After combining stock collagen solutions with the neutralizing solution, final gel concentrations were 1.5, 3.0, and 4.5  mg/mL.

#### 2.3.3 Varying crosslinking density

The degree to which the collagen matrix was crosslinked was altered through riboflavin mediated photoactivation and crosslinking [Fig. 3(b)].^38^^,^^39^ Riboflavin 5′-monophosphate sodium salt hydrate (Sigma Aldrich, St. Louis, Missouri, United States) at concentrations of 0.5 or 1 mM was added to the collagen mixture followed by 60 s of $150\;\mathrm{mW}/{\mathrm{cm}}^{2}$ UV light exposure before self-assembly. Increased crosslinking was confirmed by observing an increased linear modulus during quasi-static uniaxial tensile testing to failure in crosslinked gels compared with controls [Fig. 3(e)].

#### 2.3.4 Varying absorber density

Absorptive black pigment (Bone Black, Gamlin Pigments, Portland, Oregon, United States) was introduced to hydrogel mixtures prior to self-assembly to vary the absorber concentration [Fig. 3(c)].^37^ The total amount of light transmittance was calculated through preliminary control gels via the Beer–Lambert law and spectrophotometric measurements across the visible light spectrum^40^ [Fig. 3(f)]. Concentrations (0.2 and 0.7  mg/mL) of pigment were then added so that 33% and 67% of control transmission levels were attained, respectively.

#### 2.3.5 Experimental design

There were a total number of 216 gels made in this study. One “batch” of gels consisted of 8 control gels and 8 gels for each of the two variation levels (24 gels/batch). Each gel was measured with both rQPLI and tQPLI. DoLP colormaps were generated and AVG DoLP was calculated for each sample in each imaging mode. DoLP was the only QPLI outcome extracted for these experiments as our objective was to determine how each ECM property affects the interpretation of the sample’s birefringence.

This process was repeated for three batches for each ECM property investigated. Therefore, there were 24 gels made per variation level (control, low, and high) for a total of 72 gels/ECM property. Control gels were made in each round of fabrication to account for batch-to-batch variation in the gel making process.

#### 2.3.6 Statistical testing

A linear mixed model was used to statistically account for random effects associated with batch-to-batch variability and assess fixed effects of ECM variation on AVG DoLP values. For each group, the difference between the least square means of ECM variation minus control was calculated from the model and plotted as mean ± standard error. For each outcome measure, p values were computed for fixed effects and interactions. Where warranted by statistical results of a model’s fixed effects, *post hoc* contrast testing was performed between the model predicted least squares mean difference of the ECM variation minus the control. *Post hoc* results were adjusted with Bonferroni correction to account for multiple comparisons. All statistical analyses were performed using JMP (SAS, Cary, North Carolina, United States), and plots were produced using GraphPad Prism (San Diego, California, United States). Results were considered statistically significant for p<0.05.

### 2.4 QPLI of Deep Digital Bovine Flexor Tendons

#### 2.4.1 Sample preparation

25 mm long × 10 mm wide sections of the proximal portion of deep digital BFTs (Animal Technologies, Tyler, Texas, United States) were extracted and thinned to 1-mm-thickness on a freezing stage sledge microtome. BFTs were chosen as the model tissue as they have a highly aligned collagen microstructure that is consistent through their thickness (in the proximal region) and they have been historically used as a representative tendon tissue.^41^^,^^42^ Fiducial markers were affixed to the surface of each sample to delineate QPLI ROIs. Tissue samples (n=24) were preloaded to 0.1 N of uniaxial tension in a mechanical loading device (TestResources, Shakopee, Minnesota, United States) and prior to imaging with QPLI in both reflectance and transmission modes.

#### 2.4.2 Microstructural validation

After QPLI, a subset of BFT samples (n=3) were fixed in 10% neutral buffered formalin for 48 h before being processed and paraffin embedded for histological sectioning. 10-μm-thick sections were then stained with picrosirius red.^43^ Sections were imaged under (1) brightfield and (2) crossed linear polarizers using a Leica DMS 1000 microscope. An additional subset of tendons (n=6) was imaged with the same multiphoton configuration for SHG as mentioned with the gel microstructural imaging (ex. 880  nm/em. 440 nm). Three 100-μm-thick z-stacks (5  μm between slices) were taken for each sample, and maximum intensity projections were generated for each stack.

#### 2.4.3 Experimental design and statistical testing

AVG DoLP and STD AoP values were calculated for the ROI delineated by the fiducial markers. QPLI data (AVG DoLP and STD AoP) were statistically analyzed via a paired t-test between imaging modes, where p<0.05 was considered statistically significant. Representative color maps for DoLP and AoP at baseline for each sample in each imaging mode were also generated.

## 3 Results

### 3.1 Tissue Phantoms of Varying Collagen Fiber Alignment

The microstructure of the aligned and disorganized tissue phantoms was confirmed via SHG and OCT imaging. Expected fiber alignment was qualitatively confirmed via SHG imaging [Fig. 2(a)], with aligned gels containing more longitudinally aligned fibers compared with the disorganized gels that had a more isotropic fiber formation in the center portion. Quantitative confirmation of microstructural alignment was achieved via 3D OCT imaging and Fourier transform-based orientation analysis [Figs. 2(c)–2(e)]. Fiber concentration values (k) were significantly larger (p<0.05) for aligned gels (0.99±0.34) compared with disorganized gels (0.10±0.07) [Fig. 2(e)]. Variance (σ) values were significantly larger (p<0.05) in disorganized gels (62.7  deg±3.16  deg) than in aligned gels (37.5  deg±3.20  deg) [Fig. 2(f)].

**Fig. 4:**
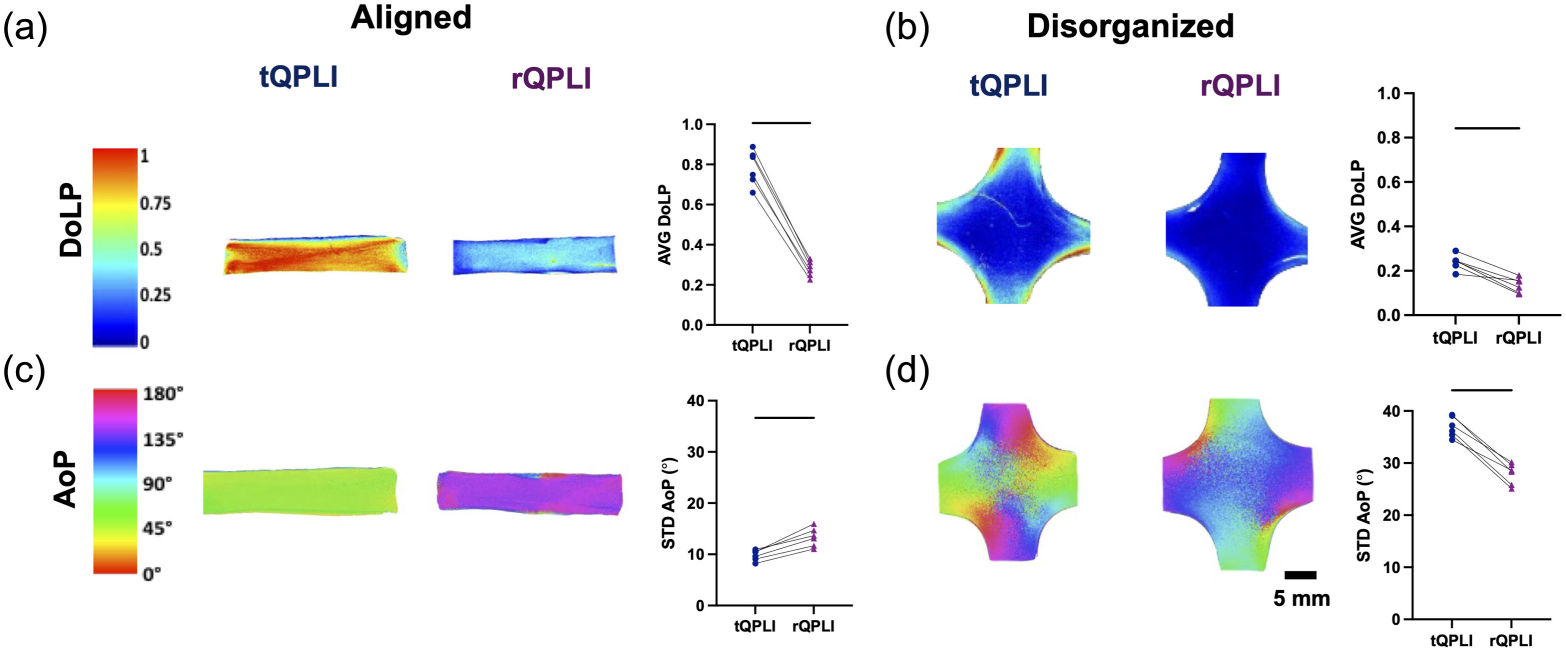
QPLI analysis of collagen tissue phantoms of varying fiber alignments. Representative color maps of [(a), (c)] aligned gels and [(b), (d)] disorganized gels. (a), (b) DoLP was quantified for the ROI indicated in the color map via AVG DoLP; (c), (d) AoP was quantified via STD AoP. Paired t-tests were used to statistically compare groups (n=6), where lines indicate p<0.05.

In both aligned and disorganized collagen gel phantoms, AVG DoLP values were significantly larger in transmission mode (aligned: 0.78±0.09; disorganized: 0.24±0.03) than in reflectance mode (aligned: 0.28±0.04; disorganized: 0.14±0.03) (p<0.05) [Figs. 4(a) and 4(b)]. A qualitative evaluation of QPLI color maps confirms these overall trends. Additionally, DoLP was decreased in disorganized gels compared with their aligned counterparts regardless of the imaging mode, as expected when considering the underlying collagen fiber alignment of each gel.

**Fig. 5:**
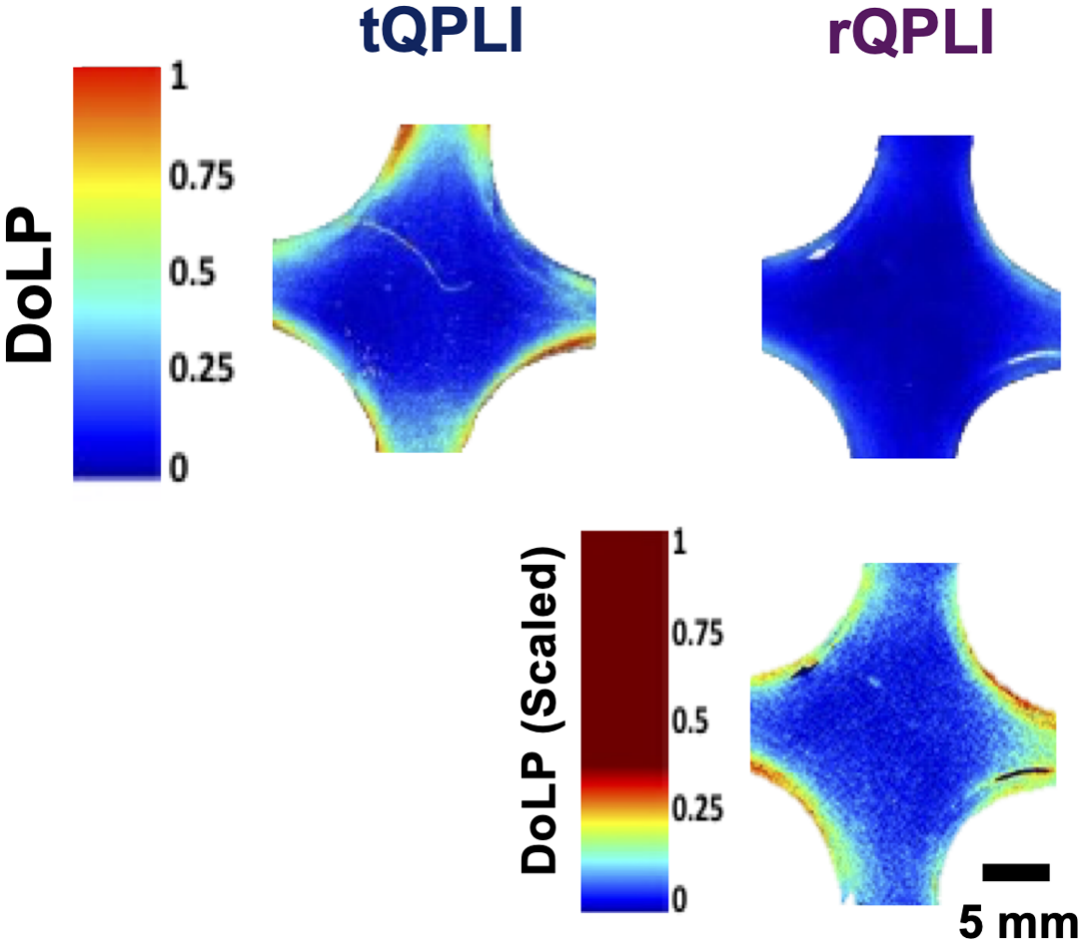
Visualization of scaling DoLP color map in rQPLI.

To determine if the decrease in DoLP in reflectance mode was a uniform decrease relative to transmission mode, scaled DoLP color maps were generated (Fig. 5). In these maps, the lookup table in reflectance mode was scaled to map from 0 to 0.25 instead of the 0 to 1 range. After scaling, the reflectance mode maps qualitatively approximated the transmission mode counterparts, indicating that the decrease in DoLP was uniform and not dependent on local collagen organization.

STD AoP values significantly increased from transmission (9.80  deg±1.04  deg) to reflectance mode (13.4  deg±1.83  deg) in the aligned gels, and conversely decreased from transmission (36.9  deg±1.96  deg) to reflectance (27.9  deg±2.05  deg) mode in the disorganized gels [Figs. 4(c) and 4(d)] (p<0.05). Additionally, STD AoP values of the disorganized gels were larger overall when compared with the aligned gels, regardless of imaging mode. This is an expected finding given the more isotropic nature of the collagen fiber alignment in the disorganized samples when compared with the anisotropic aligned gels. As AoP is indicative of collagen fiber orientation, the isotropic alignment results in a more varied AoP in the disorganized gels and thus an increased STD AoP.

**Fig. 6:**
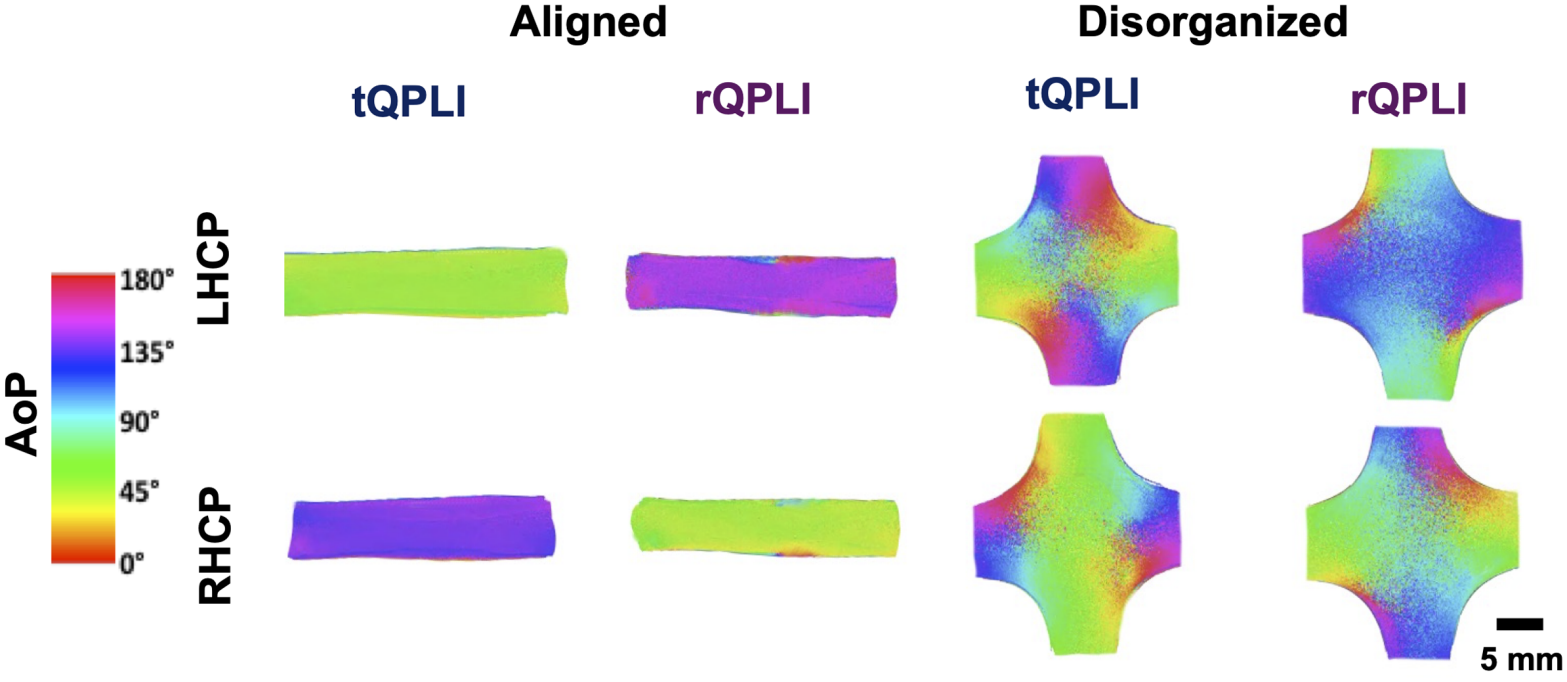
Comparison of AoP color maps of both aligned and disorganized phantoms when performing transmission and reflectance QPLI with input right-handed and left-handed circularly polarized light.

Upon inspection of the AoP color maps, there was a clear shift in values when comparing reflectance and transmission mode counterparts, which appeared to be ∼90  deg. It has previously been shown that circularly polarized photons undergo a mirror reflection and flip their helicity upon reflection but not with transmission.^44^ To investigate if this phenomenon caused the observed shift in AoP between QPLI imaging modes, gels were imaged with left-handed circularly polarized light and compared with the data obtained from the standard QPLI data acquired using right-handed circularly polarized light. By switching the handedness of input light, the AoP shifted 90 deg in both alignment types and imaging modes (Fig. 6).

### 3.2 Tissue Phantoms of Varying Extracellular Matrix Properties

**Fig. 7:**
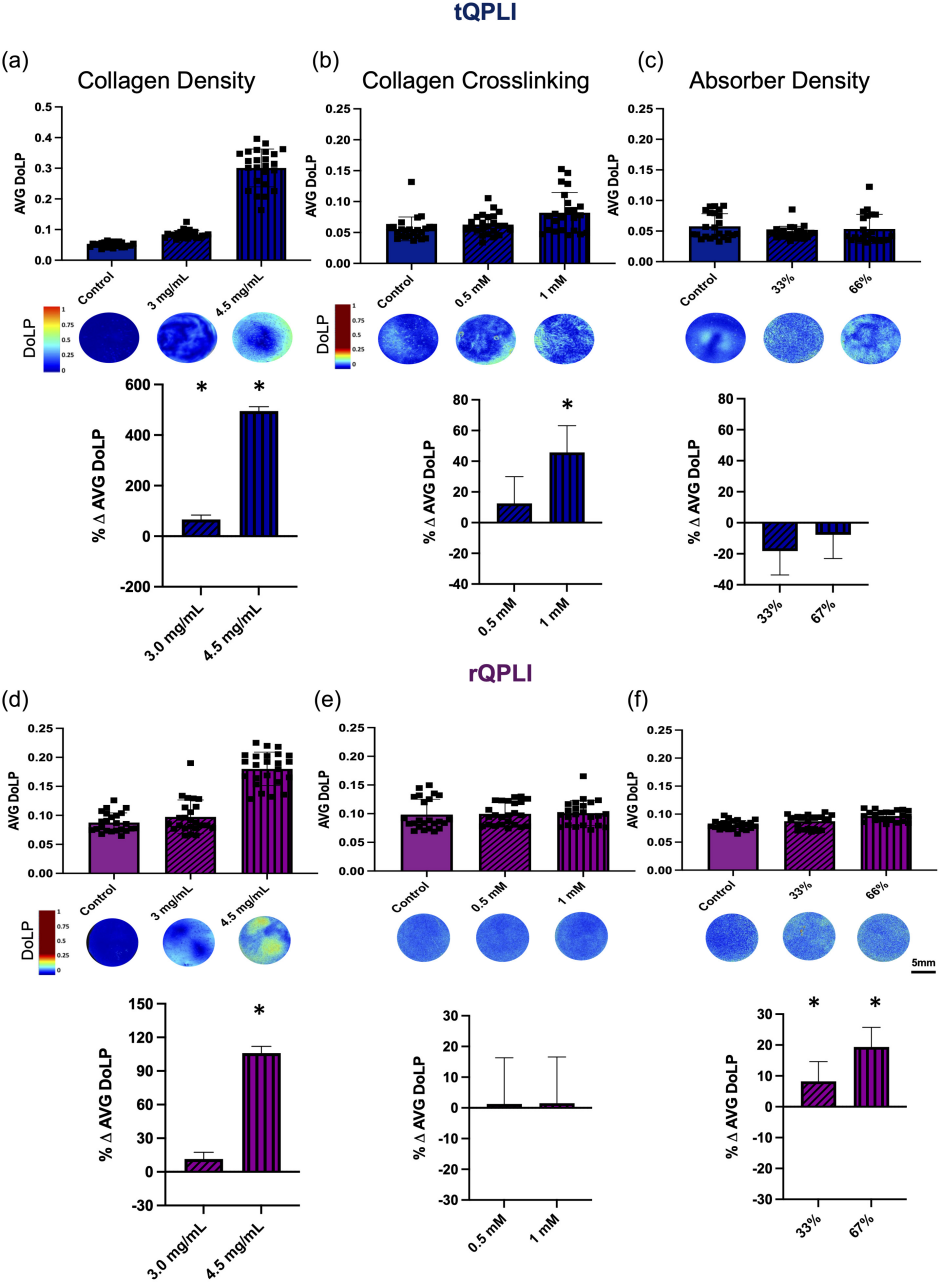
AVG DoLP of collagen gels with varying [(a), (d)] collagen density, [(b), (e)] crosslinking density, and [(c), (f)] absorber density measured with (a)–(c) tQPLI or (d)–(f) rQPLI. Top row: raw data points for each gel type along with corresponding DoLP color map for a representative gel in each group (n=24/group). Note the change in scale for tQPLI collagen density images compared with other groups. Bottom row: model predicted least square means percent difference of each ECM variation level minus control when accounting for the random effects of batch-to-batch variation. Asterisk symbol * indicates *post hoc* statistical significance (p<0.05) compared with control.

In transmission mode, increasing collagen density significantly increased the AVG DoLP of 3.0 and 4.5  mg/mL collagen gels by 66% and 495% compared with controls, respectively [Fig. 7(a)]. There was also a significant increase in AVG DoLP with increasing collagen density in rQPLI [Fig. 7(d)]. This effect was observed only in the highest (4.5  mg/mL) collagen concentration compared with controls, resulting in a 106% increase.

There was a significant effect of increasing crosslinking density on AVG DoLP in transmission mode [Fig. 7(b)]. By increasing the collagen crosslinking via 1 mM of riboflavin photoactivation, AVG DoLP values increased 46% compared with controls. There was no difference between control gels and those treated with 0.5 mM riboflavin-mediated crosslinking. There was also no effect of increasing collagen crosslinking on AVG DoLP in reflectance mode [Fig. 7(e)].

Although there was a significant difference in AVG DoLP with increasing absorber concentration in transmission mode [Fig. 7(c)], no differences in either group (33% transmissivity versus control; 67% transmissivity versus control) reached statistical significance in *post hoc* testing. In reflectance mode, increasing absorber concentration resulted in a significant increase in AVG DoLP in both concentrations by 8% and 19% compared with controls for the 33% and 67% transmissivity groups, respectively [Fig. 7(f)].

### 3.3 QPLI of Deep Digital Bovine Flexor Tendons

**Fig. 8:**
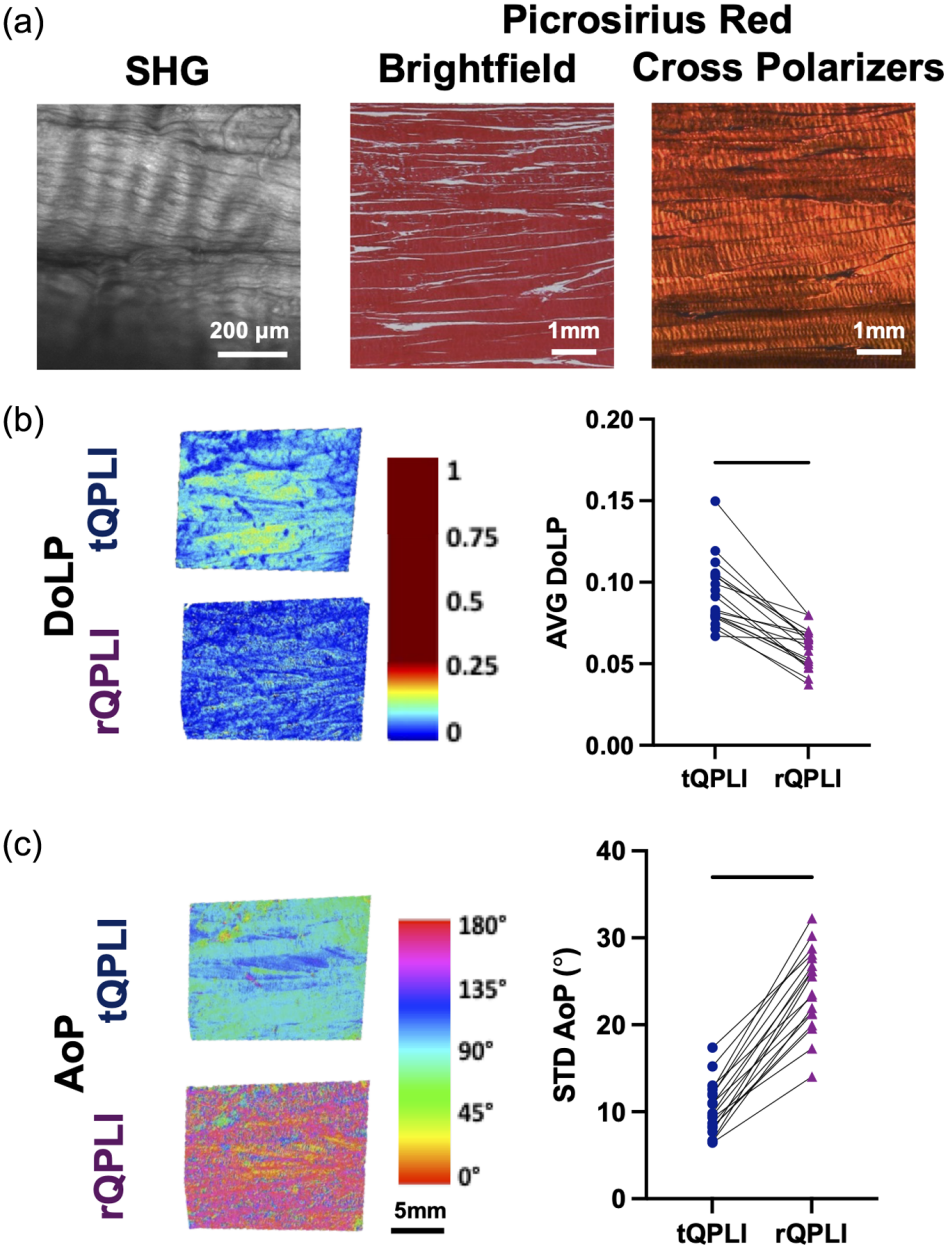
(a) Evaluation of BFT microstructure via SHG and brightfield/cross polarized imaging of picrosirius red stained histological sections. QPLI outcomes of (b) DoLP and (c) AoP for BFTs. Paired t-tests were used to statistically compare groups (n=6), where lines indicate p<0.05.

The highly aligned nature of BFTs was confirmed through SHG and histological evaluation. SHG signal obtained from BFT samples demonstrated a densely packed, highly aligned fibrous microstructure with a characteristic crimp pattern [Fig. 8(a)]. Picrosirius red staining indicated a high collagen density in BFTs, as demonstrated by intense red staining under brightfield illumination. When imaged under cross polarizers, the resulting birefringence was red-orange in color, indicative of a highly aligned, fibrous architecture [Fig. 8(a)].

AVG DoLP values were significantly larger for BFTs in transmission mode (0.09±0.02) compared with reflectance mode (0.06±0.01) (p<0.05) [Fig. 8(b)]. STD AoP values were significantly larger in reflectance mode (24.1  deg±4.73  deg) compared with transmission mode (10.3  deg±3.01  deg) [Fig. 8(c)] (p<0.05). The same 90 deg shift in AoP that was observed in the collagen tissue phantoms of different fiber alignments was observed in the representative AoP color maps of the BFTs [Fig. 8(c)].

## 4 Discussion

This study provided a systematic evaluation of how microstructural and compositional properties commonly associated with collagenous soft tissues impact outcomes acquired through division-of-focal-plane Stokes polarimetry. We also considered differences that might arise in polarimetric outcomes based on the chosen imaging geometry. With the recent commercialization of the SONY Polarsens sensor,^12^ Stokes polarimetry techniques are becoming increasingly more common in the biomedical space. Further, due to the potential for clinical impact and endoscopic integration, reflectance mode-based imaging will likely emerge as a dominant approach for *in vivo* imaging applications of complex bulk tissue.^10^ As such, it is important to have a full understanding of the many factors that influence common polarimetric outcomes of these techniques to best inform data interpretation for future studies of both basic science and translational applications.

Using a collagen gel tissue phantom model, we evaluated the sensitivity of both transmission and reflectance QPLI in the measurement of structural anisotropy. As transmission QPLI has long been used to monitor and evaluate changes in collagen fiber alignment,^6^^,^^21^ the data in this mode can act as a “ground truth” with which to evaluate the reflectance mode’s performance. Regardless of microstructure, AVG DoLP was significantly reduced in reflectance mode when compared with transmission mode (Fig. 4). However, this reduction appeared to be uniform throughout the tissue (Fig. 5), indicating that reflectance mode is sensitive to the microstructure in a similar, but scaled, manner as transmission mode. As the collected photons in reflectance mode are primarily backscattered,^5^ we hypothesize that the underlying reason for the reduction in DoLP is due to the increased number of scattering events that serve to progressively depolarize incident photons as they propagate through the tissue. If this is the case, then there is an increased number of scattering (depolarizing) events but longer cumulative distances traversed through the birefringent media (likely polarizing). Polarization-sensitive Monte Carlo models may be useful for further exploring the balance between these two interactions and may be able to improve data interpretation in more complex environments, such as native biological tissues.^45^^,^^46^

Interestingly, the previously characterized and well understood phenomenon of reflectance flipping the helicity of circularly polarized photons manifested as a 90 deg shift in AoP in our QPLI outcomes when comparing between the two imaging geometries (Fig. 6). This, similar to the DoLP magnitude decrease, was a uniform effect and was not dependent on the local microstructure. As STD AoP is typically used as an outcome for QPLI measurements, we do not expect the uniformity of this shift to influence the variance in the fiber orientations, permitting similar analysis of STD AoP values in both modes. Interestingly, we saw a microstructural-dependent effect on the STD AoP with values being larger for the aligned gels in reflectance mode but larger for the disorganized gels in transmission mode (Fig. 4). We hypothesize that this effect is due to an inherent decrease in signal-to-noise when imaging in reflectance mode. We know that reflectance mode imaging primarily collects backscattered photons whereas biological tissues typically favor forward scattering. Therefore, fewer photons return to the detector in reflectance mode than in transmission mode, leading to noisier outcomes, thus obscuring the true STD AoP of each reflectance mode image. This effect would decrease the already high STD AoP in the more disorganized phantoms and conversely increase the low STD AoP of the aligned phantoms. Further studies are needed to evaluate how noise originating from low-light conditions propagates in QPLI outcomes of DoLP and AoP to better inform data analysis of reflectance mode, particularly for applications in which light is limited such as endoscopy.^47^

Beyond collagen fiber alignment, we also investigated how other non-anisotropic ECM properties affect QPLI data, specifically the interpretation of DoLP, in both imaging modes. We hypothesized that collagen density would have the greatest effect on QPLI outcomes in both transmission and reflectance modes due to the increased birefringent material in the optical path. When using QPLI to evaluate fiber microstructure of collagen gels in transmission mode and reflectance mode, collagen density did appear to have the greatest effect on AVG DoLP. Interestingly, there was a significant increase in AVG DoLP in the highest collagen density (4.5  mg/mL) over controls for both imaging modes, but the AVG DoLP was only significantly increased in the 3.0  mg/mL group in transmission mode [Figs. 7(a) and 7(d)]. These results suggest that DoLP may be more sensitive to collagen density differences in transmission mode imaging. Higher collagen density increases the total amount of birefringent matter in the optical path but also results in an increased number of scattering events. Therefore, the outcomes of DoLP are a combination of both polarizing and depolarizing interactions with the sample. In transmission mode, the collected photons are primarily ballistic. Thus these photons are less influenced by depolarizing scattering interactions that result from increased scatterer density in the tissue, which could result in a greater sensitivity of tQPLI to increased collagen density at a lower threshold than the more scattered reflectance mode outcomes.

When considering collagen crosslinking, we hypothesized that the increased connectivity of the matrix due to the crosslinks would increase total depolarizing scattering events. However, as the length scale of the crosslinks are substantially smaller than the collagen fibrils themselves, we were unsure how large of an impact this effect would have when considering changes in AVG DoLP, which are ROI-based measurements focused on the tissue scale. AVG DoLP values were increased in the highest variation of crosslinking over controls in transmission mode, but there was no effect of crosslinking on DoLP in reflectance mode [Figs. 7(b) and 7(e)]. Again, this difference may be due in part to the difference in the nature of the photons collected in each mode: any increase in DoLP that occurs in transmission mode may be lost by the time the photons reach the sensor in reflection mode after multiple scattering events.

Finally, the absorber concentration was hypothesized to increase DoLP in both modes. By increasing the likelihood of an absorbing event, photons that make it to the detector are more likely to have had fewer tissue interactions, acting as a “gating” for photons undergoing fewer scattering events. There was little difference in DoLP in transmission mode, but DoLP was increased in reflectance mode with increasing absorber density [Figs. 7(c) and 7(f)]. This result is likely due to the “gating” effect of the absorbers having a smaller effect on the already ballistic photons in transmission mode than on the multiply scattered reflectance mode photons.

Additionally, we performed QPLI in reflectance and transmission modes on a model biological tissue of interest—the deep digital BFT—which was selected because of its high degree of fiber alignment and relative homogeneity through the depth (at least in the proximal region).^42^ Similar to the aligned collagen gels, BFTs exhibited high structural anisotropy, but were much denser, more crosslinked, and contained additional compositional elements beyond collagen.^48^ Similar to the aligned collagen gel experiments [Fig. 4(a)], a comparison of rQPLI and tQPLI in tendon showed the following: (1) a decrease in DoLP from transmission to reflectance modes, (2) a 90 deg shift in AoP between modes, and (3) an increased STD AoP from transmission to reflectance modes (Fig. 8). These similarities indicate that our findings from the collagen gel studies can be extended to other more complex, collagenous biological systems. Beyond this study, Hamilton et al.^49^ recently showed that reflectance and transmission mode polarized light microscopy techniques are equally capable of measuring collagen fiber crimp in both reflectance and transmission modes. Their findings also support the notion that both modes are similarly sensitive to the microstructure when imaging highly scattering, complex biological tissues such as the medial collateral ligament of the knee and in the annulus fibrosis of the intervertebral disc, even given differences in imaging geometry.

In summary, results of this study indicate that two dominant drivers of polarimetric outcomes in biological tissues, regardless of imaging mode, are alignment and density. The influence of both parameters is important to consider when performing imaging studies in dynamic systems. For example, when using QPLI to evaluate differences in dynamic realignment between two tendon types, the two tissue populations should have a relatively similar density to avoid confounding outcomes. Crosslinking and absorber density have a comparatively smaller effect on QPLI outcomes and as such are not likely to be able to be distinguished using QPLI but also not likely to confound evaluation of other tissue properties (e.g., collagen alignment).

This study is not without limitations. The density of collagen in the tissue phantoms was substantially less than the density of collagen in biological tissues, largely due to the inherent technical limitations of fabricating collagen hydrogels at higher densities.^50^ Additionally, the hierarchical collagen organization across length-scales, which is a hallmark of complex biological tissues, is lacking in the collagen tissue phantom structure. For example, phantoms are more immature and fibrillar than the fibrous nature of tendon; therefore, scattering would be more pronounced in biological tissues than in the phantoms. Even given these phantom limitations, the principles gleaned from the data in this study are likely to extend to more complex and mature biological tissues as indicated by the congruence of overall trends between the phantom and tendon data. Future work can employ polarization sensitive Monte Carlo models to overcome the experimental limitations of the model system and expand on the work described herein to more complex and hierarchical simulated tissues.

## 5 Conclusion

This study provides a systematic comparison of how microstructural and compositional ECM properties differentially affect polarimetric outcomes in a division-of-focal-plane-based Stokes polarimetry system operating in reflectance and transmission modes. We found that both imaging modes are sensitive to collagen alignment and density and as such should be controlled for, or at least considered, when interpreting imaging data from biological tissues. Additionally, in transmission mode, photons with fewer scattering events are collected and result in increased retention of polarization information. Conversely, in reflectance mode, there is a lower magnitude of polarization (lower DoLP) compared with transmission mode that we hypothesize is due to the increased number of depolarizing interactions associated with collecting primarily backscattered photons. Further, we have shown that the helicity flip associated with reflection of circularly polarized photons manifests as a 90 deg shift in AoP in our imaging system. Future studies carrying out polarization-sensitive Monte Carlo simulations may be a powerful tool for further investigation of light–matter interactions that differ between the two QPLI modes that cannot be easily probed experimentally, such as the interplay between polarizing (birefringent) and depolarizing (scattering) effects.

## Code, Data, and Materials Availability

Data underlying the results presented in this paper may be obtained from the authors upon reasonable request.

## References

[1] Bielajew B. J. Hu J. C. Athanasiou K. A., “Collagen: quantification, biomechanics and role of minor subtypes in cartilage,” Nat. Rev. Mater. 5(10), 730–747 (2020). 10.1038/s41578-020-0213-1 33996147 PMC8114887

[2] Parry D. A. D., “The molecular fibrillar structure of collagen and its relationship to the mechanical properties of connective tissue,” Biophys. Chem. 29(1–2), 195–209 (1988). BICIAZ 0301-4622 10.1016/0301-4622(88)87039-X 3282560

[3] Sacks M. S. David Merryman W. Schmidt D. E., “On the biomechanics of heart valve function,” J. Biomech. 42(12), 1804–1824 (2009). JBMCB5 0021-9290 10.1016/j.jbiomech.2009.05.015 19540499 PMC2746960

[4] Goth W. et al., “Optical-based analysis of soft tissue structures,” Annu. Rev. Biomed. Eng. 18, 357–385 (2016). ARBEF7 1523-9829 10.1146/annurev-bioeng-071114-040625 27420574 PMC5537381

[5] Tuchin V. V., “Polarized light interaction with tissues,” J. Biomed. Opt. 21(7), 071114 (2016). 10.1117/1.jbo.21.7.071114 27121763

[6] York T. et al., “Real-time high-resolution measurement of collagen alignment in dynamically loaded soft tissue,” J. Biomed. Opt. 19(6), 066016 (2014). JBOPFO 1083-3668 10.1117/1.JBO.19.6.066011 24972359

[7] Ghosh N. Wood M. Vitkin A., “Polarized light assessment of complex turbid media such as biological tissues using Mueller matrix decomposition,” in Handbook of Photonics for Biomedical Science, 1st ed., Tuchin V., Ed., pp. 253–282, CRC Press, Boca Raton, Florida (2010).

[8] Ghosh N. Vitkin A. I., “Tissue polarimetry: concepts, challenges, applications, and outlook,” J. Biomed. Opt. 16(11), 110801 (2011). JBOPFO 1083-3668 10.1117/1.3652896 22112102

[9] York T. et al., “Bioinspired polarization imaging sensors: from circuits and optics to signal processing algorithms and biomedical applications,” Proc. IEEE 102(10), 1450–1469 (2014). IEEPAD 0018-9219 10.1109/JPROC.2014.2342537 PMC4629637 26538682

[10] Singh M. D. Ghosh N. Vitkin I. A., “Mueller matrix polarimetry in biomedicine: enabling technology, biomedical applications, and future prospects,” in Polarized Light in Biomedical Imaging and Sensing, Ramella-Roman J. C. Novikova T., Eds., pp. 61–103, Springer, Cham (2023).

[11] Novikova T. Ramella-Roman J. C., “Is a complete Mueller matrix necessary in biomedical imaging?,” Opt. Lett. 47(21), 5549–5552 (2022). OPLEDP 0146-9592 10.1364/OL.471239 37219266

[12] Sony Semiconductor Solutions Group, “Polarization image sensor technology Polarsens™,” https://www.sony-semicon.com/en/technology/industry/polarsens.html (accessed 3 March 2023).

[13] Castile R. M. et al., “Microstructural properties and mechanics vary between bundles of the human anterior cruciate ligament during stress-relaxation,” J. Biomech. 49(1), 87–93 (2016). JBMCB5 0021-9290 10.1016/j.jbiomech.2015.11.016 26643578

[r14] Solon L. F. et al., “Mechanical properties and microstructural organization of common ulnar collateral ligament grafts: palmaris longus and gracilis tendons,” J. Orthop. Res. 40(8), 1865–1871 (2022). JOREDR 0736-0266 10.1002/JOR.25209 34786748

[r15] Smith M. V. et al., “Mechanical properties and microstructural collagen alignment of the ulnar collateral ligament during dynamic loading,” Am. J. Sports Med. 47(1), 151–157 (2019). 10.1177/0363546518812416 30495972

[r16] Skelley N. W. et al., “Differences in the microstructural properties of the anteromedial and posterolateral bundles of the anterior cruciate ligament,” Am. J. Sports Med. 43(4), 928–936 (2015). 10.1177/0363546514566192 25634908

[r17] Skelley N. W. et al., “Regional variation in the mechanical and microstructural properties of the human anterior cruciate ligament,” Am. J. Sports Med. 44(11), 2892–2899 (2016). 10.1177/0363546516654480 27456027

[r18] Wright J. O. et al., “Microstructural and mechanical properties of the posterior cruciate ligament,” J. Bone Joint Surg. Am. 98(19), 1656–1664 (2016). 10.2106/JBJS.16.00032 27707852

[19] Wu X. et al., “Comparison of high-speed polarization imaging methods for biological tissues,” Sensors 22(20), 8000 (2022). SNSRES 0746-9462 10.3390/s22208000 36298350 PMC9607302

[20] Blair M. J. Quinn K. P., “Single shot quantitative polarized light imaging system for rapid planar biaxial testing of soft tissues,” Front. Bioeng. Biotechnol. 10, 1010307 (2022). 10.3389/FBIOE.2022.1010307 36213065 PMC9532628

[21] Iannucci L. E. et al., “Optical imaging of dynamic collagen processes in health and disease,” Front. Mech. Eng. 8, 18 (2022). 10.3389/fmech.2022.855271

[22] Dayan D. et al., “Are the polarization colors of picrosirius red-stained collagen determined only by the diameter of the fibers?,” Histochemistry 93(1), 27–29 (1989). HCMYAL 0301-5564 10.1007/BF00266843 2482274

[23] López De Padilla C. M. et al., “Picrosirius red staining: revisiting its application to the qualitative and quantitative assessment of collagen type I and type III in tendon,” J. Histochem. Cytochem. 69(10), 633–643 (2021). JHCYAS 0022-1554 10.1369/00221554211046777 34549650 PMC8504258

[24] Lien C.-H. Chen Z.-H. Phan Q.-H., “Birefringence effect studies of collagen formed by nonenzymatic glycation using dual-retarder Mueller polarimetry,” J. Biomed. Opt. 27(8), 087001 (2022). 10.1117/1.JBO.27.8.087001 36452033 PMC9349470

[25] King N. O., “Biomedical applications of polarimetry,” Dissertation, Washington University, St. Louis (2020).

[26] Meitz E., “Creation of a graphical user interface for reflectance and transmission mode quantitative polarized light imaging,” Mech. Eng. Mater. Sci. Independent Study 136, 1–15 (2020). 10.7936/a4fw-pp02

[27] Powell S. B. Gruev V., “Calibration methods for division-of-focal-plane polarimeters,” Opt. Express 21(18), 21040–21055 (2013). OPEXFF 1094-4087 10.1364/OE.21.021040 24103976

[28] Gao S. Gruev V., “Bilinear and bicubic interpolation methods for division of focal plane polarimeters,” Opt. Express 19(27), 26161–26173 (2011). OPEXFF 1094-4087 10.1364/OE.19.026161 22274203

[29] Lake S. P. Barocas V. H., “Mechanics and kinematics of soft tissue under indentation are determined by the degree of initial collagen fiber alignment,” J. Mech. Behav. Biomed. Mater. 13, 25–35 (2012). 10.1016/j.jmbbm.2012.03.017 22842273 PMC3482295

[r30] Raghupathy R. et al., “Identification of regional mechanical anisotropy in soft tissue analogs,” J. Biomech. Eng. 133, 1–7 (2011). JBENDY 0148-0731 10.1115/1.4005170 PMC3705984 22010746

[r31] Jhun C.-S. et al., “Planar biaxial mechanical behavior of bioartificial tissues possessing prescribed fiber alignment,” J. Biomech. Eng. 131(8), 081006 (2009). JBENDY 0148-0731 10.1115/1.3148194 19604018 PMC3717317

[32] Chue-Sang J. et al., “Optical phantoms for biomedical polarimetry: a review,” J. Biomed. Opt. 24(3), 030901 (2019). 10.1117/1.JBO.24.3.030901 30851015 PMC6975228

[33] Iannucci L. E. et al., “Cellular and chemical gradients to engineer the meniscus‐to‐bone insertion,” Adv. Healthc. Mater. 8(7), 1800806 (2019). 10.1002/adhm.201800806 PMC6458090 30536862

[34] Kroon D. J. Slump C. H. Maal T. J. J., “Optimized anisotropic rotational invariant diffusion scheme on cone-beam CT,” Med. Image Comput. Comput. Assist. Interv. 13(3), 221–228 (2010). 10.1007/978-3-642-15711-0_28 20879403

[35] Weickert J. Scharr H., “A scheme for coherence-enhancing diffusion filtering with optimized rotation invariance,” J. Vis. Commun. Image Represent. 13(1–2), 103–118 (2002). JVCRE7 1047-3203 10.1006/jvci.2001.0495

[36] Morrill E. E. et al., “A validated software application to measure fiber organization in soft tissue,” Biomech. Model. Mechanobiol. 15(6), 1467 (2016). BMMICD 1617-7959 10.1007/s10237-016-0776-3 26946162 PMC5328598

[37] Silverio V. et al., “Dark matters: black-PDMS nanocomposite for opaque microfluidic systems,” Phys. Chem. Chem. Phys. 21(5), 2719–2726 (2019). PPCPFQ 1463-9076 10.1039/C8CP06828C 30663744

[38] Diamantides N. et al., “Correlating rheological properties and printability of collagen bioinks: the effects of riboflavin photocrosslinking and pH,” Biofabrication 9(3), 034102 (2017). 10.1088/1758-5090/aa780f 28677597

[39] Ashwin P. T. Mcdonnell P. J., “Collagen cross-linkage: a comprehensive review and directions for future research,” Br. J. Opthamol. 94(8), 965–970 (2010). 10.1136/bjo.2009.164228 19666925

[40] Wang L. V. Wu H.-I., Biomedical Optics: Principles and Imaging, John Wiley and Sons, Hoboken, New Jersey (2012).

[41] King N. O. Gruev V. Lake S. P., “Implementation of a logarithmic division-of-focal-plane polarimeter to quantify changes in collagen alignment at varying levels of illumination,” Appl. Opt. 59(26), 7813 (2020). APOPAI 0003-6935 10.1364/AO.398362 32976451

[42] Fang F. Sawhney A. S. Lake S. P., “Different regions of bovine deep digital flexor tendon exhibit distinct elastic, but not viscous, mechanical properties under both compression and shear loading,” J. Biomech. 47(12), 2869–2877 (2014). JBMCB5 0021-9290 10.1016/j.jbiomech.2014.07.026 25113805

[43] Junqueira L. C. U. Bignolas G. Brentani R. R., “Picrosirius staining plus polarization microscopy, a specific method for collagen detection in tissue sections,” Histochem. J. 11(4), 447–455 (1979). 10.1007/BF01002772 91593

[44] da Silva A. et al., “Depth selectivity in biological tissues by polarization analysis of backscattered light,” Proc. SPIE 8172, 817205 (2011). 10.1117/12.898618

[45] Novikova T. Ramella-Roman J. C., “Polarization-sensitive Monte Carlo,” in Polarized Light in Biomedical Imaging and Sensing, Ramella-Roman J. C. Novikova T., Eds., pp. 105–131, Springer, Cham (2023).

[46] Ramella-Roman J. C. Prahl S. A. Jacques S. L., “Three Monte Carlo programs of polarized light transport into scattering media: part I,” Opt. Express 13(12), 4420 (2005). OPEXFF 1094-4087 10.1364/OPEX.13.004420 19495358

[47] Qi J. Elson D. S., “Polarimetric endoscopy,” in Polarized Light in Biomedical Imaging and Sensing, Ramella-Roman J. C. Novikova T., Eds., pp. 179–204, Springer, Cham (2023).

[48] Lake S. P. et al., “Tendon and ligament tissue engineering,” in Principles of Tissue Engineering, 5th ed., Lanza R. et al., Eds., pp. 989–1005, Academic Press, Cambridge, Massachusetts (2020).

[49] Hamilton K. D. Chrzan A. J. Michalek A. J., “Reflected cross-polarized light microscopy as a method for measuring collagen fiber crimp in musculoskeletal tissues,” J. Mech. Behav. Biomed. Mater. 125, 104953 (2022). 10.1016/j.jmbbm.2021.104953 34763150

[50] Cross V. L. et al., “Dense type I collagen matrices that support cellular remodeling and microfabrication for studies of tumor angiogenesis and vasculogenesis in vitro,” Biomaterials 31(33), 8596–8607 (2010). BIMADU 0142-9612 10.1016/j.biomaterials.2010.07.072 20727585 PMC2949514

[51] Iannucci L. E. Riak M. Lake S. P., “The effect of extracellular matrix properties on polarized light-based analysis of collagen fiber alignment in soft tissues,” Proc. SPIE 11963, 1196304 (2022). PSISDG 0277-786X 10.1117/12.2614438

[52] Iannucci L. Gruev V. Lake S. P., “Comparison of transmission- and reflectance-mode quantitative polarized light imaging (QPLI) for microstructural analysis of collagenous soft tissues,” Proc. SPIE 11646, 1164609 (2021). PSISDG 0277-786X 10.1117/12.2576709

